# Depressed Cardiac Mechanical Energetic Efficiency: A Contributor to Cardiovascular Risk in Common Metabolic Diseases—From Mechanisms to Clinical Applications

**DOI:** 10.3390/jcm9092681

**Published:** 2020-08-19

**Authors:** Albert Juszczyk, Karolina Jankowska, Barbara Zawiślak, Andrzej Surdacki, Bernadeta Chyrchel

**Affiliations:** 1 Students’ Scientific Group at the Second Department of Cardiology, Jagiellonian University Medical College, 2 Jakubowskiego Street, 30-688 Cracow, Poland; albert.juszczyk@gmail.com (A.J.); karoliina.jankowska@gmail.com (K.J.); 2 Department of Cardiology and Cardiovascular Interventions, Intensive Cardiac Care Unit, University Hospital, 2 Jakubowskiego Street, 30-688 Cracow, Poland; zawislak.barbara@gmail.com; 3 Department of Cardiology and Cardiovascular Interventions, University Hospital, 2 Jakubowskiego Street, 30-688 Cracow, Poland; surdacki.andreas@gmx.net; 4 Second Department of Cardiology, Jagiellonian University Medical College, 2 Jakubowskiego Street, 30-688 Cracow, Poland

**Keywords:** external cardiac work, cardiac mechanical energetic efficiency, noninvasive methods, cardiovascular risk, insulin resistance, diabetes, metabolic syndrome, hypertension

## Abstract

Cardiac mechanical energetic efficiency is the ratio of external work (EW) to the total energy consumption. EW performed by the left ventricle (LV) during a single beat is represented by LV stroke work and may be calculated from the pressure–volume loop area (PVLA), while energy consumption corresponds to myocardial oxygen consumption (MVO_2_) expressed on a per-beat basis. Classical early human studies estimated total mechanical LV efficiency at 20–30%, whereas the remaining energy is dissipated as heat. Total mechanical efficiency is a joint effect of the efficiency of energy transfer at three sequential stages. The first step, from MVO_2_ to adenosine triphosphate (ATP), reflects the yield of oxidative phosphorylation (i.e., phosphate-to-oxygen ratio). The second step, from ATP split to pressure–volume area, represents the proportion of the energy liberated during ATP hydrolysis which is converted to total mechanical energy. Total mechanical energy generated per beat—represented by pressure–volume area—consists of EW (corresponding to PVLA) and potential energy, which is needed to develop tension during isovolumic contraction. The efficiency of the third step of energy transfer, i.e., from pressure–volume area to EW, decreases with depressed LV contractility, increased afterload, more concentric LV geometry with diastolic dysfunction and lower LV preload reserve. As practical assessment of LV efficiency poses methodological problems, De Simone et al. proposed a simple surrogate measure of myocardial efficiency, i.e., mechano-energetic efficiency index (MEEi) calculated from LV stroke volume, heart rate and LV mass. In two independent cohorts, including a large group of hypertensive subjects and a population-based cohort (both free of prevalent cardiovascular disease and with preserved ejection fraction), low MEEi independently predicted composite adverse cardiovascular events and incident heart failure. It was hypothesized that the prognostic ability of low MEEi can result from its association with both metabolic and hemodynamic alterations, i.e., metabolic syndrome components, the degree of insulin resistance, concentric LV geometry, LV diastolic and discrete systolic dysfunction. On the one part, an increased reliance of cardiomyocytes on the oxidation of free fatty acids, typical for insulin-resistant states, is associated with both a lower yield of ATP per oxygen molecule and lesser availability of ATP for contraction, which might decrease energetic efficiency of the first and second step of energy transfer from MVO_2_ to EW. On the other part, concentric LV remodeling and LV dysfunction despite preserved ejection fraction can impair the efficiency of the third energy transfer step. In conclusion, the association of low MEEi with adverse cardiovascular outcome might be related to a multi-step impairment of energy transfer from MVO_2_ to EW in various clinical settings, including metabolic syndrome, diabetes, hypertension and heart failure. Irrespective of theoretical considerations, MEEi appears an attractive simple tool which couldt improve risk stratification in hypertensive and diabetic patients for primary prevention purposes. Further clinical studies are warranted to estimate the predictive ability of MEEi and its post-treatment changes, especially in patients on novel antidiabetic drugs and subjects with common metabolic diseases and concomitant chronic coronary syndromes, in whom the potential relevance of MEE can be potentiated by myocardial ischemia.

## 1. Introduction

The prevalence of cardiovascular (CV) diseases remains high in the modern world, regardless of well-recognized modifiable CV risk factors. Despite rigorous treatment goals and lifestyle modifications recommended by practice guidelines, these targets are not achieved in the majority of patients with most common CV risk factors, such as hypertension, obesity and type 2 diabetes (DM), constituting major public health concerns. In order to improve primary prevention strategy, there is a need to optimally use easily accessible parameters to ameliorate risk stratification and enhance implementation of aggressive prophylactic measures. In this review, we focus on a non-invasive index of cardiac mechanical efficiency which, despite its simplicity, has proved its predictive ability in prospective clinical studies with regard to the risk of major adverse CV events or new-onset heart failure (HF).

## 2. Cardiac Mechanical Energetic Efficiency—Basic Concepts

**Figure 1:**
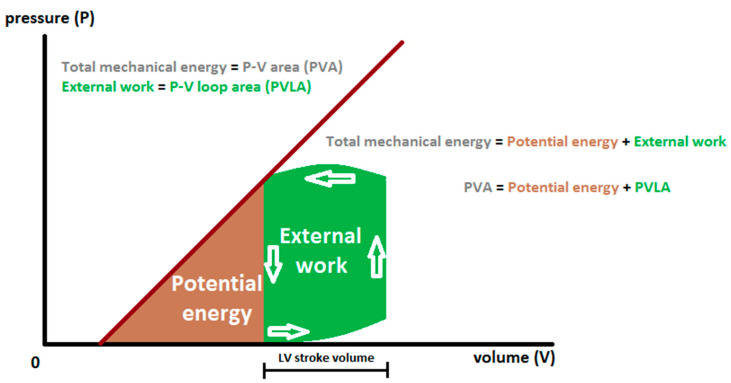
Diagrammatic representation of left ventricular pressure–volume relations. Total mechanical energy (represented by pressure–volume area (PVA)) is the sum of potential energy (PE) and external work (EW), corresponding to pressure–volume loop area (PVLA) [3,4,7].

**Figure 2:**
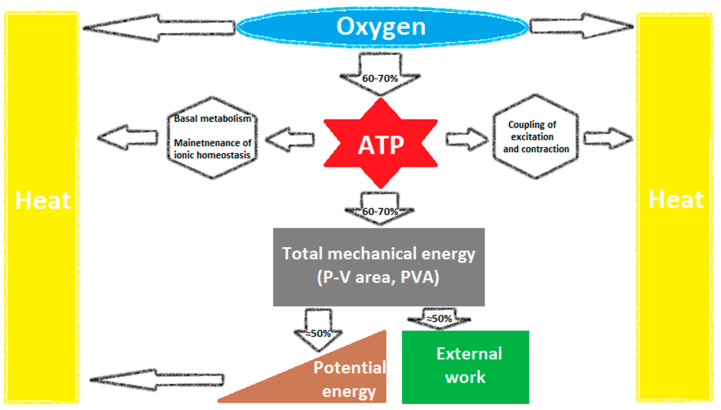
Multi-step energy transfer from myocardial oxygen consumption to external work (EW): 1—from oxygen consumption to ATP synthesis; 2—from ATP split to pressure–volume area (PVA) (reflecting total mechanical energy); 3—from PVA to EW [3]. ATP: adenosine triphosphate.

Mechanical energetic efficiency of a system can be universally defined as the ratio of external work performed by the system to the total energy consumption [1,2,3,4]. In heart physiology, external work (EW) performed by the left ventricle (LV) during a single beat is represented by stroke work (SW) and may be calculated from the pressure–volume loop area (PVLA) (Figure 1). Energy consumption corresponds to myocardial oxygen consumption (MVO_2_) assuming a constant energy equivalent per amount of oxygen metabolized. MVO_2_ can accurately be measured by coronary sinus catheterization as a product of myocardial blood flow and the coronary arteriovenous difference in oxygen content, expressed on a per-beat basis. Early studies estimated mechanical efficiency of the human LV at about 20–25% [2,4,5,6] (Figure 1 and Figure 2).

Total mechanical efficiency is a joint effect of the efficiency of energy transfer at several sequential stages [3,8] (Figure 2). The first step, from oxygen consumption to adenosine triphosphate (ATP), reflects the yield of oxidative phosphorylation (i.e., phosphate-to-oxygen ratio) and depends on the predominant fuel used as energy source and the efficacy of mitochondrial ATP synthesis. Its efficiency averages 60–70% and the remaining energy is converted to heat [3,8] (Figure 2).

The second step, from ATP split to pressure–volume area (PVA) (Figure 2), represents the proportion of the energy liberated during ATP hydrolysis which is converted into total mechanical energy (TME) by myosin ATPase (about 60–70%), which corresponds to 30–50% of MVO_2_ [3,8]. The residual energy is used for non-mechanical activities of contraction and basal metabolism, including excitation–contraction coupling, maintenance of ionic environment (especially Ca^2+^-ATPase in the sarcoplasmic reticulum and sarcolemmal Na^+^-K^+^-ATPase), various protein phosphorylation pathways and synthetic purposes, and its majority is converted to heat [3,8] (Figure 2). 

TME generated per beat—represented by PVA—consists of EW (corresponding to PVLA) and potential energy (PE) (Figure 1). PE is the energy needed to develop tension and alter the geometry of elastic elements during isovolumic contraction [9], its part is stored in the LV wall at end-systole and—as early-diastolic restoring forces—accounts for LV elastic recoil and diastolic suction [3]. The efficiency of the third step of energy transfer, i.e., from PVA to EW (represented by the proportion of PVLA relative to PVA) (Figure 1 and Figure 2), averages 50‒70% and depends on loading conditions and contractile state [3,4,8].

## 3. Myocardial Mechano—Energetic Efficiency Index (MEEi)—A Simple Surrogate Measure of LV Mechanical Efficiency

Coronary sinus catheterization is rarely performed and the assessment of MVO_2_ with positron emission tomography poses methodological problems [8], which profoundly limits the assessment of LV mechanical efficacy in the clinical practice. In brief, indices of the turnover of ^11^C-labelled acetate and ^15^O-labelled water (from inhaled ^15^O_2_) enter equations depicting estimates of MVO_2_, while LV mass (LVM) and volumes may be derived from echocardiography, magnetic resonance or nuclear imaging, and brachial blood pressure is measured by a sphygmomanometer [8,10,11,12]. However, beyond sophisticated equipment, methodological constraints of this approach include limited reliability of the conversion of a semiquantitative index of myocardial oxidative metabolism from the ^11^C-acetate-based method to equivalents of absolute units, the variability of the metabolic fate of ^11^C-acetate, tracer spill-over artifacts and the need of arterial blood sampling [8]. Although the ^15^O_2_-based method appears to be a golden standard of MVO_2_ estimation, its feasibility is profoundly limited by technical problems related to the multiple tracer usage and challenging data analysis [8].

Therefore, in 2009, De Simone et al. [9] proposed a simple method of the estimation of myocardial mechano-energetic efficiency (MEE) from routine echocardiography. 

According to this concept [8], stroke work (SW) can be obtained by the following Equation (1):

SW = SBP × SV [mmHg × mL] = SBP × SV × 0.014 [Cj](1)

where SBP is systolic blood pressure, SV is stroke volume in mL measured during a standard echocardiographic evaluation, and 0.014 is a conversion factor used to change units from mmHg × mL to cJ (centijoules), traditionally—albeit incorrectly—termed “gram-meters” [9,13].

MVO_2_ may be approximated by calculating the so-called double product, i.e., SBP multiplied by heart rate (HR)—Equation (2):

MVO_2_ ≈ SBP × HR (2)

Thus, MEE is presented as a simplified formula—Equation (3):

MEE = SW/MVO_2_ ≈ (SBP × SV)/(SBP × HR) = SV/HR (3)

Following the initial concept by De Simone et al. [8], MEE is expressed in mL/s, with HR value “adjusted” to a reference HR of 60 beats/min. Accordingly, rationalizing this traditional unit of measurement, MEE would represent the amount of blood pumped by the LV during 1 s at a HR of 60 beats/min.

Since a linear positive correlation between MEE and LVM was found in the initial report, MEE index (MEEi) was computed as MEE divided by LVM [9]. Obviously, MEEi decreases with increasing HR, decreasing SV and increasing LVM. De Simone et al. [9] proposed that lower MEEi at higher HR can correspond to the fact that during isovolumic contraction, which occurs more frequently when HR accelerates, does not generate EW, so that a higher proportion of energy is dissipated as heat, thus resulting in lower efficiency.

## 4. Low MEEi—A Predictor of Short- and Long-Term CV Events

In a report from the Campania Salute Network Registry [7], 12,353 hypertensive subjects without prevalent CV disease and with preserved ejection fraction (EF > 50%) (mean age, 52 ± 13 years; 44% women; obesity prevalence, 26%; DM prevalence, 10%) were followed for a median of 31 months (interquartile range, 2–64 months) regarding the incidence of 3-point major adverse CV events (MACE) (i.e., acute myocardial infarction, stroke, and sudden death) or composite CV events, including 3-point MACE, HF requiring hospitalization, coronary revascularization, angina and atrial fibrillation.

During the follow-up, 136 incident 3-point MACE and 359 composite CV events were observed. The association of low MEEi at baseline (i.e., ≤0.29 mL/s per g, corresponding to the lowest quartile of its distribution) with the risk of either 3-point MACE or composite CV events remained significant upon adjustment for demographical characteristics, baseline and follow-up blood pressure, antihypertensive treatment, obesity, DM and LVH [7]. Although there was no significant interaction between low MEEi and the presence of LVH, adjustment for low MEEi slightly reduced the excessive hazard of MACE and composite CV events attributable to LVH, suggesting that a part of detrimental LVH effects might result from depressed MEEi [7]. Additionally, although the use of antihypertensive drugs was more frequent in those with low MEEi, however, the adjustment for antihypertensive medication and follow-up blood pressure did not change the risk estimates, which prompted the hypothesis that the myocardial damage associated with low MEEi at the baseline of the follow-up might be irreversible [7]. 

In a population-based cohort, 1912 American Indians participating in the Strong Heart Study (SHS) (mean age, 59 ± 8 years; 64% women; obesity prevalence, 51%; DM prevalence, 40%) free of prevalent CV disease and with preserved EF were followed for a median of 9.9 years (interquartile range, 9.3‒10.4 years) [14]. Low MEEi at baseline (i.e., ≤0.34 mL/s per g, corresponding to the lowest quartile of its distribution) predicted incident HF (126 events) upon adjustment for demographical factors, CV risk factors, antihypertensive therapy, LV diastolic dysfunction and interval myocardial infarction as a competing risk event [14]. In the subjects with low MEEi, the cumulative risk of HF after 100 months was about 5-fold higher (~5%) compared to the remainder (~1%). Low MEEi at baseline was 2-fold more frequent in patients with versus without incident HF during the follow-up (47% vs. 24%) [14].

In the seminal study performed in newly diagnosed, untreated patients with hypertension and without CV disease, De Simone et al. [9] compared 56 hypertensive subjects with MEEi below the 10th percentile of the distribution with 250 remaining hypertensives. The former subjects exhibited increased SBP and HR, depressed LV midwall fractional shortening at unchanged end-systolic LV wall stress (consistent with discrete LV systolic dysfunction despite preserved EF), more concentric LV geometry and higher excess of LVM relative to estimated afterload, corresponding to increased prevalence of a so-called inappropriate LV hypertrophy (LVH) [9]. In both aforementioned prospective studies of subjects free of CV disease and with preserved EF [7,14], these findings were confirmed and supplemented with a higher prevalence of impaired LV relaxation in subjects with low MEEi. In both presented studies [7,14], despite progressive rises in the prevalence of coexistent hemodynamic abnormalities across decreasing MEEi quartiles, MEEi predictive ability was virtually restricted to its lowest quartile, which suggests that the prognostic effect appears only below a certain threshold value.

The hemodynamic alterations are likely to decrease the efficiency of the third step of energy transfer from oxygen consumption to EW, i.e., from TME (PVA) to EW. In particular, the proportion of EW relative to TME (i.e., PVLA relative to PVA) decreases with depressed LV contractility (via higher PE) [7], increased afterload (via higher relative rises in PE than EW) [4], as well as in concentric LV geometry and diastolic dysfunction (via lower EW due to lower SV and limited LV preload reserve).

## 5. Association of Low MEEi with Metabolic Risk Factors

In addition to hemodynamic alterations, reports from three independent cohorts suggested the association of low MEEi with metabolic risk factors, such as obesity, insulin resistance (IR), metabolic syndrome and DM [7,14,15,16,17], which may be a consequence of a common underlying mechanism. In the fasting state, the main energy source of cardiomyocytes is free fatty acids (FFA) oxidation. Under exercise conditions or in advanced HF, the relative use of glucose or lactate increases, which is metabolically more efficient [18]. Admittedly, partially inconsistent results were reported in clinical studies and in animal experimental models of HF, which was to some degree attributable to confounding effects of coexistent diseases and levels of circulating FFA and ketone bodies [18]. Notably, the transition from compensated LVH to HF in Dahl salt-sensitive rats was accompanied by “metabolic remodelling”, reflected by a depressed phosphocreatine-to-ATP ratio, decreased gene expression of enzymes related to FFA oxidation and mitochondrial function, and also reduced expression of glycolytic enzymes and pyruvate dehydrogenase, coupling glycolysis to glucose oxidation [19]. It is noteworthy that dichloroacetate, known to up-regulate pyruvate dehydrogenase, activated the pentose phosphate pathway, increased the phosphocreatine-to-ATP ratio, improved LV function and prevented HF development in that experimental model [19]. 

IR hinders the usage of glucose and lactate as energy sources and favors FFA utilization, which is associated with impaired energetic efficiency due to a lower yield of ATP per mole of oxygen consumed (i.e., phosphate-to-oxygen ratio) [3,18], which may contribute to decreased MEEi in insulin-resistant states [15,16].

A total of 3128 non-diabetic participants of the SHS and the Strong Heart Family Study free of CV disease, with available data on fasting glucose and insulin concentrations, were divided into four groups based on the quartiles of HOMA-IR (Homeostatic Model Assessment for Insulin Resistance) index, an estimate of the magnitude of IR [15]. With increasing HOMA-IR index, MEEi progressively decreased, which was highly significant and independent of kinship, demographical data, major CV risk factors and inflammation markers [15]. Hence, IR could be considered as a powerful independent correlate of low MEEi.

In another cohort, 12,503 participants of the previously mentioned Campania Salute Network Registry (of whom women represented 44%), with available glucose levels, were divided into four groups according to the presence of DM or metabolic syndrome [16]. Both DM and metabolic syndrome were associated with lower MEEi, with the lowest MEEi value when DM and metabolic syndrome coexisted [16]. Beyond this association, decreased MEEi was also independently associated with male gender, higher SBP, increased body-mass index, concentric LV geometry, more use of renin-angiotensin axis antagonists, diuretics and calcium-channel blockers, and less use of beta-blockers [16].

Recently, Fiorentino et al. [17] reported a decreased MEEi in 363 non-diabetic patients with ultrasound-defined non-alcoholic fatty liver disease, which was entirely mediated by the degree of IR, reflected by either HOMA-IR index or a surrogate measure of hepatic IR.

Mechanistically, the joint contribution of IR and impaired glucose tolerance to low MEEi might be attributed to the competition between FFA and glucose/lactate for oxidation via the Randle cycle-related pathways [18]. In insulin-resistant states, insulin-dependent inhibition of the hormone-sensitive triglyceride lipase is depressed, which results in increased FFA mobilization from adipose tissue. Increased uptake of FFA (either bound to plasma albumin or contained in very low-density liporoteins or chylomicrons triacylglycerols after the lipoprotein lipase-mediated hydrolysis) by cardiomyocytes favors FFA beta-oxidation which generates more mitochondrial acetyl-coenzyme A (acetyl-CoA) [18]. Acetyl-CoA inhibits mitochondrial pyruvate dehydrogenase, which uncouples glycolysis from pyruvate oxidation. In addition, increased condensation of acetyl-CoA and oxaloacetate generates citrate which enters the cytosol via the tricarboxylate carrier and inhibits phosphofructokinase [18]. Moreover, a higher myocardial uptake of FFA activates also pathways associated with FFA turnover associated with the generation of lipid intermediates, such as ceramides and diacylglycerols (DAG) which further impair insulin sensitivity with consequent depression of glucose uptake and glycolysis [18]. DAG contribute to IR via protein kinase C activation, while long-chain ceramides induce IR through inhibition of protein kinase B (Akt)/phosphoinositide 3-kinase signaling, increase oxidative stress and cause mitochondrial dysfunction in cardiomyocytes, in part by a direct effect on the electron transport chain [20,21,22,23].

When IR is paralleled by hyperinsulinemia, these abnormalities are partially compensated by higher insulin levels. In contrast, when IR is accompanied by developing beta-cell dysfunction, the IR-dependent shift of myocardial metabolism in favor of FFA is potentiated, which decreases the rate of ATP production at a given rate of oxygen consumption. Thus, the additive contribution of IR and beta-cell dysfunction to depressed MEE might explain joint effects of the metabolic syndrome (associated with IR) and DM (presumably with coexistent beta-cell dysfunction) on MEEi in the Campania Salute Network Registry [16].

Finally, the proposed concept is consistent with independent additive associations of incident HF with both reduced glucose disposal rate (assessed the euglycemic insulin clamp technique, the golden standard method for IR quantification) and fasting intact proinsulin levels and 2-h post-challenge glucose concentrations (estimates of beta-cell dysfunction) in 1187 community-based elderly subjects without prevalent HF participating in the Uppsala Longitudinal Study of Adult Men [24]. Notably, these relations were maintained after adjustment for baseline DM status, overall and truncal obesity variables, established CV risk factors and interim myocardial infarction over a median follow-up of about 9 years [24]. 

Beyond the lower maximal achievable ATP yield per oxygen molecule for FFA than glucose [3,18], excessive use of FFA instead of glucose under insulin-resistant conditions can also contribute to depressed MEE through the previously mentioned detrimental effects of ceramide on mitochondria related to the inhibition of the electron transport chain (i.e., electron leak) [20] and decreased mitochondrial membrane potential (i.e., proton leak) [23], as well as elevated consumption of ATP for non-contractile purposes, such as maintenance of ionic homeostasis [3,18].

In mitochondria, electrons from glucose and FFA pass through the electron transport chain, which generates a proton gradient across the inner mitochondrial membrane, a driving force for ATP synthesis. If electrons leave the electron transport chain before oxygen is reduced to water, superoxide is generated. The electron leak is the predominant mechanism of the formation of reactive oxygen species (ROS) in mitochondria, accounting for the vast majority of cellular ROS production [25]. On the basis of hydrogen peroxide emission, mitochondrial ROS was earlier estimated at about 1–2% of the total oxygen consumed by the mitochondria [25,26]. Nevertheless, real ongoing ROS generation presumably represents an even higher percentage of the total oxygen consumption flux, taking into account hydrogen peroxide scavenging by the thioredoxin and glutathione systems in the mitochondrial matrix [27], which also decreases the yield of oxidative phosphorylation per oxygen molecule.

Recently, an acute inhibition of malonyl-CoA decarboxylase (MCD) (potentiating the malonyl-CoA-dependent inhibition of FFA entry to the mitochondria) resulted in a switch from FFA to glucose utilization in normal rat hearts, and improved EF and SV in post-infarction failing hearts [28]. Additionally, in the experimental model of post-infarction HF, chronic MCD blockade increased EF and EW at a similar ATP formation rate, which indicates better energetical efficiency [28]. Furthermore, chronic MCD inhibition downregulated ceramide synthesis and prevented an increase in the generation of protons in post-infarction HF, as estimated by the comparison of glycolysis rate and glucose oxidation rate [28]. Accordingly, it can be proposed that—in order to prevent cellular acidification, induced by the uncoupling of glycolysis and glucose oxidation associated with increased FFA use in that HF model—the Na/H exchanger is activated with consequent Na^+^ influx and consequent Ca^2+^ accumulation via the Ca/Na exchanger operating in the reverse mode [18,28]. Consequently, these changes stimulate ATP-utilizing ionic pumps in the sarcoplasmic reticulum and plasma membrane to re-establish Na^+^ and Ca^2+^ homeostasis, so that less ATP is available for contraction [18,28].

Thus, an increased reliance of cardiomyocytes on the oxidation of FFA at the expense of glucose, typical for IR, is associated not only with a lower yield of ATP per oxygen molecule (owing to both the lower phosphate-to-oxygen ratio for FFA than glucose and concomitant mitochondrial dysfunction), but also lower mechanical energy per ATP molecule, which decreases energetic efficiency of the first and second step of energy transfer from oxygen consumption to EW.

## 6. Relevance of MEEi—Pros and Cons

Regardless of the above-summarized evidence, it can also be argued that the predictive value of MEEi might—at least in part—actually result from a combination of three established outcome predictors, i.e., increased HR, decreased SV and higher LVM into a single index. Obviously, low MEEi is likely to reflect the amplified prognostic ability of the integrated parameters. Indeed, De Marco et al. [29] described independent associations of elevated HR and depressed SV index (SVI) with future incidence of HF in 2885 SHS participants with preserved EF and without prevalent HF. Moreover, these relations emerged in a multivariate analysis, adjusted for intervening myocardial infarction as a competing risk event. Notably, in a univariate analysis, patients with low SVI had a higher risk of incident myocardial infarction (12% vs. 8%), but similar HF risk (8% vs. 7%) over a mean 12-year follow-up [29].

These findings may be interpreted as a consequence of the association between low SVI and low LVM, as pointed out by the authors [29], so that opposite relations of these prognosticators with HF incidence cancelled each other in univariate analysis; nonetheless, the predictive value of SVI was revealed by multivariate approach. In agreement with this concept, the independent association between LVH and HF risk (also corrected for interim myocardial infarction) was described in a similar, yet not identical, group of 1912 SHS participants with preserved EF and without prevalent HF or atherosclerotic CV disease during an average follow-up of 9 years [14]. In addition, the presence of LVH was associated with an increased risk of major adverse CV events (including HF development) and all-cause death in large cohorts of patients with hypertension [30] or symptomatic severe aortic stenosis undergoing transcatheter or surgical valve replacement [31].

Nevertheless, LVM and LVH were not included in the multivariate analysis in the former report [29] of SHS Investigators, while neither SV nor HR were considered as candidate covariates in the latter study [14]. Notably, HR was higher in those with lower SV [29] or MEEi [14] in the SHS cohorts, and higher HR was also associated with decreased MEEi in the previously cited paper by De Simone et al. [9]. As HR is the denominator in the MEEi formula, MEEi decreases with increasing HR, a well-recognized CV risk factor.

Accordingly, based on these reports [9,14,29], an added predictive value of MEEi regarding HF development cannot be unequivocally confirmed beyond the association of low MEEi with decreased SV, increased LVM and accelerated HR.

Admittedly, it can be argued that MEEi calculation represents a too simplified approach to the estimation of MEE. First, mean arterial pressure, not SBP, is frequently used in the formulas for LV stroke work [8,11,12]. Second, even if this limitation would bedisregarded, the derivation of LV stroke work from SV and blood pressure is associated with a considerable bias because the pressure–volume loop is not a rectangle, and its real-time shape can only be assessed by a pressure–volume conductance catheter placed in the LV [8]. In particular, a curvilinear pressure–volume relationship in the diastolic filling phase and systolic ejection phase can lead to overestimation or underestimation of LV stroke work under different loading conditions, as elegantly reviewed by Knaapen et al. [8]. Third, the double product is only a rough surrogate measure of MVO_2_ and better noninvasive indices of myocardial oxygen demand would be more appropriate, such as a so-called triple product, i.e., the product of heart rate, LV mass and end-systolic circumferential LV wall stress [32]. Notably, Devereux et al. [32] demonstrated an independent association of lower triple product with lower risk of adverse CV events in 905 LIFE trial participants with treated hypertension and electrocardiographic LVH.

On the other hand, MEEi seems to be a stronger predictor of incident HF than SVI. On multivariate analysis, low MEEi was associated with a more pronounced increase of the risk of incident HF (mean hazard ratio, 1.61) [14] compared to low SVI (mean hazard ratio, 1.38) [29]. Additionally, this effect was observed despite the fact that low MEEi was defined as an MEEi below the 25th percentile of its distribution among SHS subjects [14], which was a less rigorous criterion than that for SVI (below the 19th percentile) in a similar SHS population [29]. Therefore, a stronger association with HF risk could be expected for SVI than MEEi, while an opposite was observed, which supports the concept of the clinical relevance of MEEi.

Irrespective of detailed mechanisms underlying its prognostic capability in the given patient, MEEi elegantly integrates SV, HR and LVM into a simple estimate. Thus, this index also reflects mechanistic pathways, underlying predictive value of low SV and high LVM in hypertensive and diabetic patients. These pathways include restrictive LV remodeling, diastolic dysfunction and interrelated abnormalities, such as subtle circumferential and longitudinal systolic dysfunction despite preserved EF [33,34,35,36,37,38], coronary microvascular dysfunction with depressed coronary flow reserve [39,40,41,42,43], and inappropriate LVH relative to afterload [44,45,46], which are usually not assessed in the real-world clinical practice.

These abnormalities—jointly contributing to LV concentric geometry, diastolic and subtle systolic dysfunction—decrease the efficiency of the third step of energy transfer from oxygen consumption to EW, as discussed previously. In addition, the association of low MEEi with metabolic risk factors [7,14,15,16,17], conditions with a potential to impair the efficiency of the first two steps of the energy transfer, extends the mechanistic basis of the prognostic value of low MEEi with regard to new-onset HF.

## 7. Implications for Future Research—Treatment Effects on MEE

Metabolic effects have been implicated in the antianginal activity of trimetazidine and perhexiline in patients with ischemic heart disease [18]. Trimetazidine is a competitive inhibitor of 3-ketoacyl-CoA thiolase, catalyzing the final step of each cycle of FFA beta-oxidation with the resultant formation of acetyl-CoA [18]. Consequently, a lower generation of acetyl-CoA is a likely cause of improved coupling between glycolysis and glucose oxidation with subsequent prevention of intracellular acidosis and Na^+^ and Ca^2+^ overload [18]. Perhexiline also decreases myocardial FFA beta-oxidation, owing to the inhibition of carnitine palmitoyltransferase-1, thereby attenuating mitochondrial FFA uptake [18]. However, the simultaneous cellular accumulation of phospholipids predisposes to hepatic steatosis/necrosis and peripheral neuropathy, which has considerably limited the use of the drug [18].

Among established drugs for HF, beta-blockers were reportedly shown to improve cardiac metabolism, which could contribute to their recognized energy-sparing effect, primarily linked to a drop in heart rate and myocardial contractility. Early studies suggested that possible direct metabolic effects of beta-blockerst could result from decreased availability of circulating FFA for myocardial uptake and beta-oxidation through the inhibition of hormone-sensitive lipase in adipocytes [18].

Metformin, a first-line antidiabetic drug, is known to inhibit mitochondrial complex I and up-regulate 5′-adenosine monophosphate-activated protein kinase, a pivotal energy sensor in mammalian cells [47,48,49,50]. Metformin was recently shown to improve cardiac efficiency in non-diabetic insulin-resistant patients with systolic HF, by reducing MVO_2_ (estimated by means of ^11^C-acetate positron emission tomography) at unchanged LV stroke work [51].

Cardiac metabolism has recently regained attention with the introduction of novel antidiabetic drugs. Among multiple possible pleiotropic effects of sodium-glucose cotransporter-2 inhibitors (SGLT2i), increased concentrations of ketone bodies, termed a “thrifty substrate” [52] or “superfuel” [53], were proposed as a potential contributor to the ability of the drugs to prevent HF and HF-related events. However, the net effect of ketone bodies on cardiac metabolism remains controversial [53]. First, the phosphate-to-oxygen ratio, albeit higher for ketone bodies than FFA, is nevertheless lower compared to glucose. Second, ketone bodies, glucose and FFA compete with each other for mitochondrial oxidative energy metabolism [53]. Third, regardless of being utilized as an energy substrate, ketones can also directly modulate various signaling pathways, i.e., via mitochondrial protein hyperacetylation with consequent IR and mitochondrial dysfunction [53]. Fourth, exposure of isolated failing or control working hearts to high ketone concentrations up-regulated ketone oxidation rate, overall ATP production and oxygen consumption at unchanged cardiac work, which corresponded to the lack of improvement in cardiac efficiency [54,55]. Finally, cardiac EW, ATP production, glucose and FFA oxidation were increased in isolated hearts from empagliflozin-treated diabetic animals [56]. However, these changes were not accompanied by any changes in ketone oxidation rates or cardiac efficiency [56], which indicates that myocardial metabolic effects of SGLT2i may be mediated by the mechanisms other than enhanced utilization of ketone bodies, e.g., possible inhibition of myocardial Na/H exchanger [57] with consequent protective effect on mitochondrial respiratory function [58]. Intriguingly, in an experimental model of post-infarction HF in non-diabetic pigs, empagliflozin increased myocardial ATP content, improved LV systolic function and efficiency with a concomitant switch from glucose utilization toward ketone bodies, FFA and branched-chain amino acids [59]. 

The impact of therapeutic interventions on MEEi was rarely investigated in clinical studies. In patients with mild systolic HF and metabolic syndrome, MEEi increased after 12 months in patients randomized to an aldosterone receptor antagonist on the top of optimal therapy, which was associated with improved LV diastolic function and decreased levels of B-type natriuretic peptide compared to placebo [60]. Recently, in a small group of aortic stenosis subjects, a surrogate estimate of MVO_2_ dropped and the EW-to-TME ratio increased immediately after transcatheter aortic valve implantation (TAVI), and over-median post-TAVI rises in the ratio (normalized to the individualized estimate of MVO_2_) were associated with a lower risk of all-cause death over an approximately 3-year follow up [61].

## 8. Conclusions

The association of low MEEi with adverse CV outcome might be related to multi-step impairment of energy transfer from MVO_2_ to external work in various clinical setting, including metabolic syndrome, diabetes, hypertension and HF. Irrespective of theoretical considerations, MEEi appears an attractive simple tool which could improve risk stratification in hypertensive and diabetic subjects for primary prevention purposes. Further clinical studies are warranted to estimate the predictive ability of MEEi and its post-treatment changes, especially in patients on novel antidiabetic drugs and subjects with common metabolic diseases and concomitant chronic coronary syndromes, in whom the potential relevance of MEE can be potentiated by myocardial ischemia.
